# Characteristics of cast Ti_53.3-x_Nb_10_Zr_10_Ni_10_Co_10_Fe_6.7_B_x_ compositionally complex alloys

**DOI:** 10.1038/s41598-024-78854-w

**Published:** 2024-11-29

**Authors:** Mostafa Alshafey, H. Megahed, Shimaa El-Hadad, Hisashi Sato, Lamiaa Z. Mohamed

**Affiliations:** 1 Mechanical Design and Production Engineering Department, Faculty of Engineering, Cairo University, Giza, 12613 Egypt; 2 Central Metallurgical Research and Development Institute (CMRDI), P.O. 87, Helwan, Egypt; 3 Department of Engineering Physics, Electronics and Mechanics, Nagoya Institute of Technology, Nagoya, 466-8555 Japan; 4 Mining, Petroleum, and Metallurgical Engineering Department, Faculty of Engineering, Cairo University, Giza, Egypt

**Keywords:** TiNbZr compositionally complex alloys, Boron, Microstructure, Hardness, Wear resistance, Mechanical engineering, Materials science

## Abstract

In the current investigation, elemental boron was added to form a series of Ti_53.3-x_Nb_10_Zr_10_Ni_10_Co_10_Fe_6.7_B_x_ Compositionally Complex Alloys (CCAs). Alloying was done via vacuum arc melting in amounts of 0.0, 5.3, and 10.6 at.%. From the thermodynamic parameters, adding B to the base alloy increased the system’s entropy. The microstructure of the prepared CCAs was characterized using scanning electron microscopy, transmission electron microscopy, and X-ray diffraction (XRD). The mechanical properties of CCAs as related to microstructure were assessed. According to XRD results, B-based intermetallic phases were obtained in the prepared CCAs, which were binary as Ti_3_B_4_ and ZrB_2_ and ternary as FeNbB and Nb_3_Co_4_B_7_. These intermetallic phases notably provided strengthening effects to the B-added alloys. Ti_48_Nb_10_Zr_10_Ni_10_Co_10_Fe_6.7_B_5.3_ CCA showed the most homogenous microstructure obtained by the arc melting process. Adding B increased Young’s modulus from 141 GPa (without B) to 195 GPa and 260 GPa with 5.3 and 10.6 at.%B, respectively. Hardness also increased from 502 to 606 HV with 5.3 at.% B and to 648 HV with 10.6 at.%B. Accordingly, the wear resistance increased with B addition where 10.6 at.%B sample showed the lowest wear rate among the other conditions. However, 5.3 at.% B was nominated as the optimum addition amount due to its notable microstructure homogeneity.

## Introduction

In conventional alloys, the mechanical properties of the alloy are determined based on the properties of a single element. For example, the carbon content of ferritic steels with low levels of carbon is the key component that determines their mechanical properties^1–3^. For solid solution strengthening, carbon is used as an interstitial solute element; nevertheless, the bulk of the strength is contributed by iron. Similarly, in Ti- or Al-based alloys, the proportion of Ti/Al makes up the alloy properties. Titanium alloys lose their strength when tempered at 350–550 °C, negatively affecting their performance in several high-temperature applications. High entropy alloys (HEAs) were developed in 2004 to solve similar problems for several high-temperature alloys^4^.

HEAs are generally defined as alloys of five or more elements combined in equi- or near-equi-molar proportions^5^. Being solid solutions alloys, HEAs grasped the attention due to their high strength, good thermal stability, and corrosion resistance^6–9^. The thermal stability of HEAs was proved to be controllable by incorporating elements that form stable phases. Pradhan et al.^10^ replaced Ni in the Cantor alloy (CoCrFeMnNi)) with Mo (σ-phase forming elements) and interestingly obtained four phases that were stable up to the melting point. Refractory HEAs were designed to combine high strength with superior thermal stability^11^. Kumar et al.^12^ in their study on MoNbTa0.5Ti0.5W refractory HEAs observed that the elemental segregation of W and Ta (in the dendritic region) and that of Mo, Nb, and Ti in the (inter-dendritic area), led to solid solution hardening that raised the microhardness of these regions to 4.8 and 5.75 GPa respectively.

Because there is no one dominant component, the hardness and strength of HEAs are determined by their structural kinds rather than the HEAs themselves^13^. Ni with its FCC crystal structure induces a more lattice distortion effect in HEAs, which prevents crystal dislocation and improves strain hardening. On the other hand, Fe is an α-phase with a BCC crystal at room temperature but transforms to FCC-phase at 910 °C, aiding in solid solution evolution and strength^14^. Based on this concept, BCC-HEAs may be chosen if high-yield strengths are sought; on the other hand, FCC-HEAs are preferred when low-yield strengths (high plasticity) are desired. Combining the two different structures in two-phase high-energy alloys (HEAs) leads to the creation of balanced properties, such as high strength and exceptional ductility, called the cocktail effect of HEAs. The blending of several traits creates the “cocktail” effect. According to Cao et al.^15^, the ‘cocktail’ effect in HEAs was induced mainly by local dislocation brought on by the metastable crystal structure and lattice distortion brought on by substantial atomic size variations^16^.

Recent investigations on Ti-based HEAs have focused on varied compositions and processing techniques, revealing intriguing phase stability and mechanical properties. These studies have explored different combinations of Ti with transition metals, aiming to optimize the alloys’ microstructure and enhance their performance. However, most of the work done on HEAs is in the laboratory stage, and not generally employed in the industry due to the difficulty of obtaining only solid solutions in the alloy structure^17^. Besides, intermetallic phases are frequently needed for strengthening purposes to achieve the service requirements. Some research works^18,19^ developed multicomponent alloys that contain intermetallic phases and were deviated from HEAs. According to Manzoni and Glatzel^18^, CCAs are sometimes obtained accidentally if the preparation conditions of HEAs (composition, cooling rate, etc.) are not satisfied. On the other hand, some researchers prepared CCAs intentionally to enhance the mechanical properties via intermetallic formation^20^. It has been reported that incorporating p-block elements such as nitrogen (N), carbon (C), and boron (B) resulted in the development of new high-entropy materials such as high-entropy nitrides, carbides, and borides^21,22^. Small B additions to Ti alloys improved their processibility thus leading to dramatic cost reduction^23^. B is entirely soluble in the Ti- liquid phase but has negligible solubility in the solid phase and hence eliminates the embrittlement problem caused by other interstitial elements such as H, C, or O^24,25^. Nb, Zr, Fe, Co, and Ni can also be added as alloying elements to the Ti matrix to improve their properties^26–29^. Ti-Fe and Ti-Fe-Co alloys with high mechanical properties and good ductility were produced at a low cooling rate following pre-melting in an arc furnace^30^. Ti-13Nb-13Zr, for example, is a near beta-Ti alloy which is disadvantaged by its low hardness when used for hip endoprostheses head and acetabulum applications^31^.

**Fig. 1:**
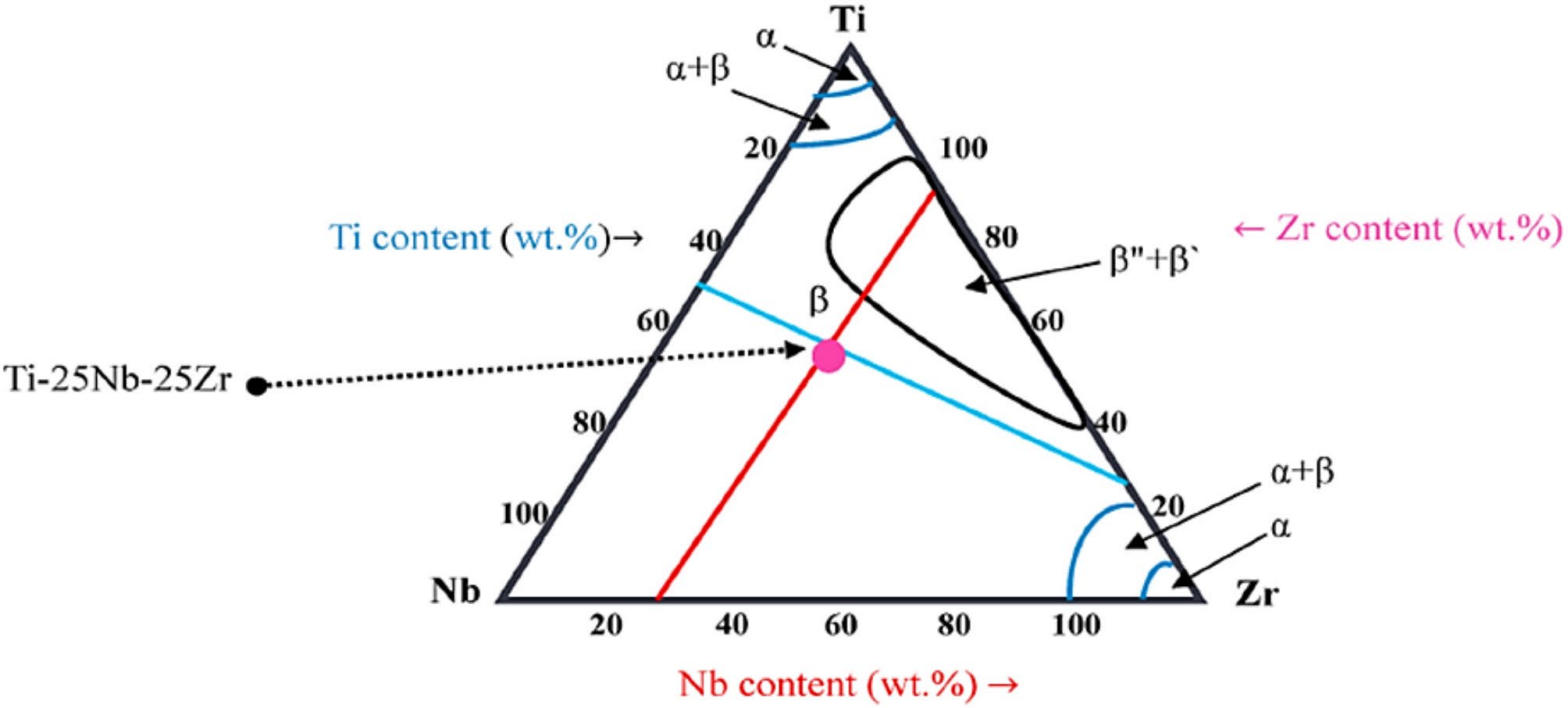
Ternary diagram of Ti-25Nb-25Zr^32^.

The TiNbZr group is classified as a beta-phase alloys based on its elemental composition. Ti, Nb, and Zr exhibit a beta-like crystal structure at room temperature, characterized by a close-packed hexagonal arrangement of atoms^32^. The ternary system of Ti25Nb25Zr alloy is shown in Fig. 1^32^. Ti-Nb and Ti-Zr-Nb alloys exhibit significantly higher hardness compared to cp-Ti grade 2, while their elastic modulus is either lower or comparable (e.g., Ti-12Nb). As a result, they are expected to offer improved wear resistance and longer service life as biomedical materials^33^. The Ti-Nb-Zr alloy system is widely regarded as one of the top candidates for developing safe and highly competitive biomedical alloys. However, their broader use is restricted by relatively low strength, hardness, and wear resistance. A significant enhancement in strength can be achieved by creating metal matrix composites reinforced with high-strength ceramic particles. Among the most promising reinforcing materials is TiB fiber, which has excellent compatibility with the titanium matrix, a similar coefficient of thermal expansion, and good thermal stability^34^. Another study added TiB_2_ as a reinforcement to TiNbZr alloy to increase the strength of the alloy^35^.

The design of HEAs is guided by four key principles: the high-entropy effect, lattice distortion, sluggish diffusion, and the “cocktail” effect^36^. The design approach of Ti-base HEAs, such as Ti_53.3-x_Nb_10_Zr_10_Ni_10_Co_10_Fe_6.7_B_x_, involves a strategic selection of elements based on targeted properties like mechanical strength, corrosion resistance, and stability at high temperatures. Titanium forms the base, offering excellent strength-to-weight ratio and corrosion resistance, while Nb improves mechanical strength and toughness, especially at lower temperatures and corrosion resistance^37,38^. Zirconium enhances both corrosion resistance and strength, stabilizing the β-phase for better ductility^37^. Nickel also stabilizes the β-phase, improving corrosion and oxidation resistance at higher temperatures. Cobalt contributes to wear resistance and elevated temperature strength, while iron enhances toughness, although it must be carefully balanced to avoid brittle phases. In titanium alloys without B, typical intermetallic phases such as Ti_2_Ni can form, known for their high strength and hardness but often exhibit limited ductility and toughness. While these intermetallics enhance overall strength at elevated temperatures, they can lead to brittleness under stress or impact. The presence of B often leads to the formation of boride phases that can further enhance mechanical properties while maintaining a high degree of entropy, that is crucial for the overall performance and reliability of the alloy in demanding environments. These elements are chosen to enhance the mechanical properties of Ti-base HEAs^39^.

Boron solubility is less than 1 at.% in beta-Ti and alpha-Ti^40^. As a result, B is largely insoluble in titanium, leading to formation of TiB particles^30,39^. Boron addition typically leads to grain refinement through several mechanisms, such as promoting nucleation during solidification by altering solidification pathways, which results in finer grains. Additionally, the formation of boride compounds can act as grain boundary pinning agents, preventing the growth of larger grains during processing and heat treatments. Boron also modifies the thermodynamic properties of the alloy, stabilizing finer microstructures during solidification and cooling processes. As boron content increases, it influences the solidification behavior of the alloy by promoting the formation of boride compounds, which refine the microstructure. This refined structure typically features smaller, more equiaxed grains due to heightened nucleation rates during solidification. Conversely, lower B levels may lead to diminished nucleation and the formation of larger, columnar grains, particularly under conditions that favor directional solidification. Moreover, the cooling rate during solidification plays a critical role; higher B levels can promote rapid cooling, while lower levels may lead to slower cooling rates, facilitating the development of larger, columnar grains^41^.

Many Ti-based CCAs still face challenges related to specific mechanical properties under extreme conditions. However, much of the research has focused on conventional CCA designs, which creates a gap in exploring novel compositions that leverage titanium’s inherent advantages while introducing new functionalities. The present investigation seeks to address this gap by developing a novel Ti-based CCAs system that incorporates a unique combination of alloying elements. By systematically analyzing the microstructural characteristics and mechanical properties of this new alloy, this paper aims to provide insights that could lead to enhanced performance in demanding applications^36^. In the current research, B was added to enhance the mechanical properties of novel Ti_53.3-x_Nb_10_Zr_10_Ni_10_Co_10_Fe_6.7_B_x_ CCAs via the intended formation of intermetallic phases while maintaining the high entropy effect. The thermodynamic parameters of the CCAs were calculated to evaluate the entropy level. Also, the microstructures were examined using SEM, EDX, and TEM. The mechanical properties of the prepared CCAs were assessed using Young’s modulus evaluation by pulse-echo method, hardness measurements, and wear property testing. The results were then related to the microstructure characteristics, including the B-based intermetallic phases.

## Experimental work

### Materials

Ti_53.3-x_Nb_10_Zr_10_Ni_10_Co_10_Fe_6.7_B_x_ CCAs were prepared by the vacuum arc melting method (VAM). Ti, Nb, Zr, Ni, Co, Fe, and B were added as pure metals. Model (1250 LHD-China) vacuum arc-melting furnace prepared the different compositions. This furnace has three units: a control unit, a melting chamber, and a chiller. In VAM, the process starts by positioning the pure metallic pieces into the crucible (water-cooled) and then evacuating the melting chamber to avoid the reactivity of the constituting elements with air, especially Ti, which has a high oxygen affinity. Once the required vacuum degree is reached, an inert gas (argon) is injected into the chamber. A high voltage is applied between the tungsten electrode and the copper crucible. When the gun touches the crucible, it creates a spark that ignites an intense arc, thus melting the charge. By increasing the applied current, the temperature can be boosted to around 3000 C^42^. The melt is electromagnetically stirred to ensure proper mixing of different constituents, producing homogeneous samples. Finally, a disc sample of 10 mm thickness and 80 mm diameter was obtained. Three alloys, Ti_53.3-x_Nb_10_Zr_10_Ni_10_Co_10_Fe_6.7_B_x_ (where x is 0.0, 5.3, and 10.6 at.% B), were obtained. The melting chamber and the prepared sample are presented in Fig. S1.

### Investigation of Ti_53.3-x_Nb_10_Zr_10_Ni_10_Co_10_Fe_6.7_B_x_ CCAs

#### Microstructure characterization

The Ti_53.3-x_Nb_10_Zr_10_Ni_10_Co_10_Fe_6.7_B_x_ CCAs samples were cut and ground using SiC emery papers up to grid 1200 then polished by alumina paste of 0.3 µm, and finally etched using 7%HF, 8%HNO_3_ and the rest is distilled water for microstructure characterization. The microstructure of Ti_53.3-x_Nb_10_Zr_10_Ni_10_Co_10_Fe_6.7_B_x_ CCAs was studied using an SEM (Quanta FEG 250 with field emission gun, FEI − Netherlands) and the elemental distribution was detected using SEM & EDX system (JEOL Ltd. JSM-7100F-Thermo Fisher Scientific K.K. Ultra-Dry). A transmission electron microscope (TEM) model (JEOL Ltd. JEM-2100Plus) was also used to investigate the alloy crystallinity further.

#### Phase identification

The phase identification was performed to recognize the formation of the phases in the different CCAs using an X-ray diffractometer model (D8 Discover, Bruker) with Cu Kα radiation λ equal to 1.54 A, 40 kV.

#### Hardness measurements

The influence of B addition on the hardness of CCAs was evaluated using a 10 kg Vickers hardness tester (model Zwick/Roell (ZHU250)). An average of five readings was considered.

#### Wear resistance

**Fig. 2:**
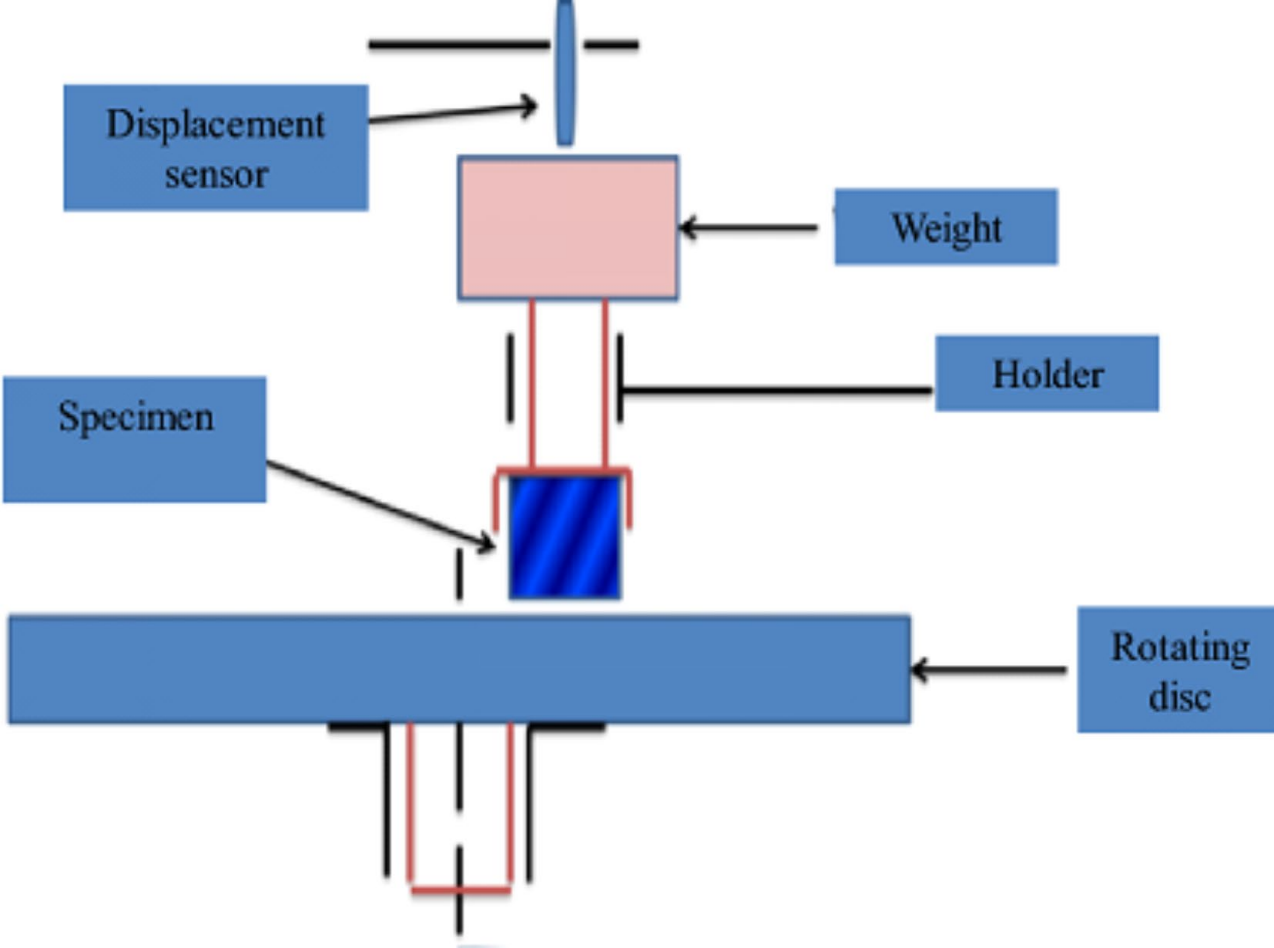
Schematic of the dry wear test setting.

The wear test was performed using a load of 10 kg and a speed of 1 m/s with 0.9 km sliding displacement (T-01 M Pin-on-Disk testing machine). A schematic illustration of the wear test is shown in Fig. 2. The coefficient of friction (COF) was calculated by using Eq. (1), and the wear rate (WR) using Eq. (2)^43^.

1 $${\text{COF}} = \frac{F}{N}$$

where F is the tangential friction force, and N is the normal applied load^44^

2 $${\text{WR}} = \frac{{{\Delta w}}}{Ls}$$

where Δ w is the weight loss measured in grams, and Ls is the sliding distance in kilometers.

#### Young’s modulus

Young’s modulus was measured by pulse-echo method at 5 MHz frequency and room temperature using sensitivity balance model Mettler H 72, capacity 160 g, readability 0.1 mg. with ultrasonic flaw detector model USM3. The Young’s modulus was calculated using several equations from Eqs. (3) to (7). Longitudinal modulus (L) was calculated by Eq. (3), where the shear modulus (G) by Eq. (4) and the bulk modulus (K) by Eq. (5). The Young’s modulus (E) by Eq. (6) and Poisson’s ratio (σ) by Eq. (7)^43^.

3 $$L=\rho {v}_{l}^{2}$$

4 $$G=\rho {v}_{s}^{2}$$

5 $$\text{K}=L-(4/3)\text{G}$$

6 $$\text{E}=2(1+\sigma )\text{G}$$

7 $$\sigma =(L-2\text{G})/2(\text{L}-\text{G})$$

## Results and discussions

### Alloy design and thermodynamic parameters of the prepared CCAs

Similar to HEAs, the classical Hume-Rothery rules^45,46^ can be used to determine the empirical thermodynamic parameters that rely on the mixing enthalpy ($\Delta {H}_{mix}$), the mixing entropy ($\Delta {S}_{mix}$), the atomic size mismatch ($\delta$) and electro-negativities to comprise the solid-solution creation between elements in the CCAs. The atomic size difference (d) is essential to the physical characteristic of a solid solution state. Zhang et al.^45^ defined the parameter ($\delta$) instead of (d) to reflect the overall impact of the atomic size mismatch (in multi-component alloys) between the primary elements. According to Wang et al.^47^, the parameter of δ was thought to be related to the strain energy but without any physical verification. The parameter δ can be defined as follows:

8 $$\delta \%= \sqrt{\sum_{i=1}^{n}{c}_{i } {(1-\frac{{r}_{i}}{\sum_{i=1}^{n}{c}_{i }{r}_{i}})}^{2}}$$

where ${c}_{i}$ and ${r}_{i}$ are the atomic fraction of the $i$ element of the individual alloy component, respectively. The mixing enthalpy of the medium entropy and high entropy alloys with $n$ elements were determined by Takeuchi et al.^48^ from the following:

9 $$\Delta {H}_{mix}= \sum_{i=1, i\ne 1}^{n}{\Omega }_{ij}{c}_{i} {c}_{j}$$

where ${\Omega }_{ij}$ denotes the regular melt-interaction parameter between the ^i^th and the ^j^th elements, and cj denotes the ^j^th molar fraction of the $j$ component, which ${\Omega }_{ij}$ denotes estimated from the following:

10 $${\Omega }_{ij}=4 \left({\Delta H}_{AB}^{mix}\right)$$

where ${\Delta H}_{AB}^{mix}$ denotes the binary alloys’ mixing enthalpy due to the Miedema model^48^. The mixing entropy of the disordering solid solution is determined by^44^:

11 $$\Delta {S}_{mix}= -R \sum_{n=1}^{n}{c}_{i}\text{ln}{c}_{i}$$

where R denotes the universal gas constant that has a value of 8.314 J/mol K. To further characterize the structural relaxation behavior, another criterion with a thermodynamics-originated parameter $\Omega$ that can be written as follows^49^:

12 $$\Omega = {T}_{m} \frac{\Delta {S}_{mix}}{\left|\Delta {H}_{mix}\right|}$$

where ${T}_{m}$ represents the average melting temperature for the medium and high entropy systems computed using the following equation as a first-order estimation^50^:

13 $${T}_{m}= \sum_{i=1}^{n}{C}_{i} {({T}_{m})}_{i}$$

The *ΔH*_*mix*_, *ΔS*_*mix*_, Ω and *δ*% values exist in Table 1. The $\delta \%$ values are higher for all investigated alloys than 6.6%, which is not a stable solid solution according to Zhang’s criteria^45^. The mixing enthalpy of all alloys is between −16.80 to −18.97 kJ/mole. Also, the mixing entropy is increased from 11.31 for 0 at.%B, to 13.39 for 5.3 at.%B, and to 14.16 for 10.6 at.%B. The $\Omega$ is related to $\Delta {H}_{mix}$ and $\Delta {S}_{mix}$. Formation of solid solution when larger than 1.1, and creation of intermetallic components when less than one. Thus, from Table 1, $\Omega$ enhances the solid solution phase formation. When t $he \Delta {S}_{mix }/R$ is greater than or equal to 1.5, the alloy can be considered as high entropy CCA, (HECCA), and when it is within 1 and 1.5 it is named medium entropy CCA (MECCA). The value of $\Delta {S}_{mix }/R$ is equal to 1.36 for Ti_53.3_Nb_10_Zr_10_Ni_10_Co_10_Fe_6.7_ alloy. Thus, this alloy is MECCA For Ti_48_Nb_10_Zr_10_Ni_10_Co_10_Fe_6.7_B_5.3_ and Ti_42.7_Nb_10_Zr_10_Ni_10_Co_10_Fe_6.7_B_10.6_ alloys, $\Delta {S}_{mix }/R$ is equal to 1.61 and 1.70, respectively. Thus, these alloys are HECCAs. According to Guo et al.^51^, it is important to calculate the valence electron concentration (VEC) to estimate the stability of the phases in HEAs. In HEAs, if VEC ≤ 7.30, then the BCC structure is dominant, while the dual phase structure HEAs (BCC + FCC) have (7.30 < VEC < 7.72), and those where (VEC ≤ 7.30), are FCC-HEAs. Table 1 shows that the total VEC of all the investigated samples is lower than 7.3, indicating that the main phase is BCC, whose stability increases by adding the elemental B.

**Table 1:** Calculated thermodynamics parameters of ME- and HE-CCAs.

Alloys	$\delta , \%$	$\Delta {H}_{mix}$ KJ/mole	$\Delta {S}_{mix}$ KJ/mole	VEC	${T}_{m}$(°C)	$\Omega$	$\Delta {S}_{mix }/R$
Ti_53.3_Nb_10_Zr_10_Ni_10_Co_10_Fe_6.7_	7.838	− 18.9727	11.3198	5.468	2043.2	1.2191	1.3615
Ti_48_Nb_10_Zr_10_Ni_10_Co_10_Fe_6.7_B_5.3_	10.570	− 18.1222	13.3866	5.415	2050.4	1.5146	1.6101
Ti_42.7_Nb_10_Zr_10_Ni_10_Co_10_Fe_6.7_B_10.6_	12.601	− 16.8027	14.1621	5.362	2057.6	1.7342	1.7034

### Phase identification

**Fig. 3:**
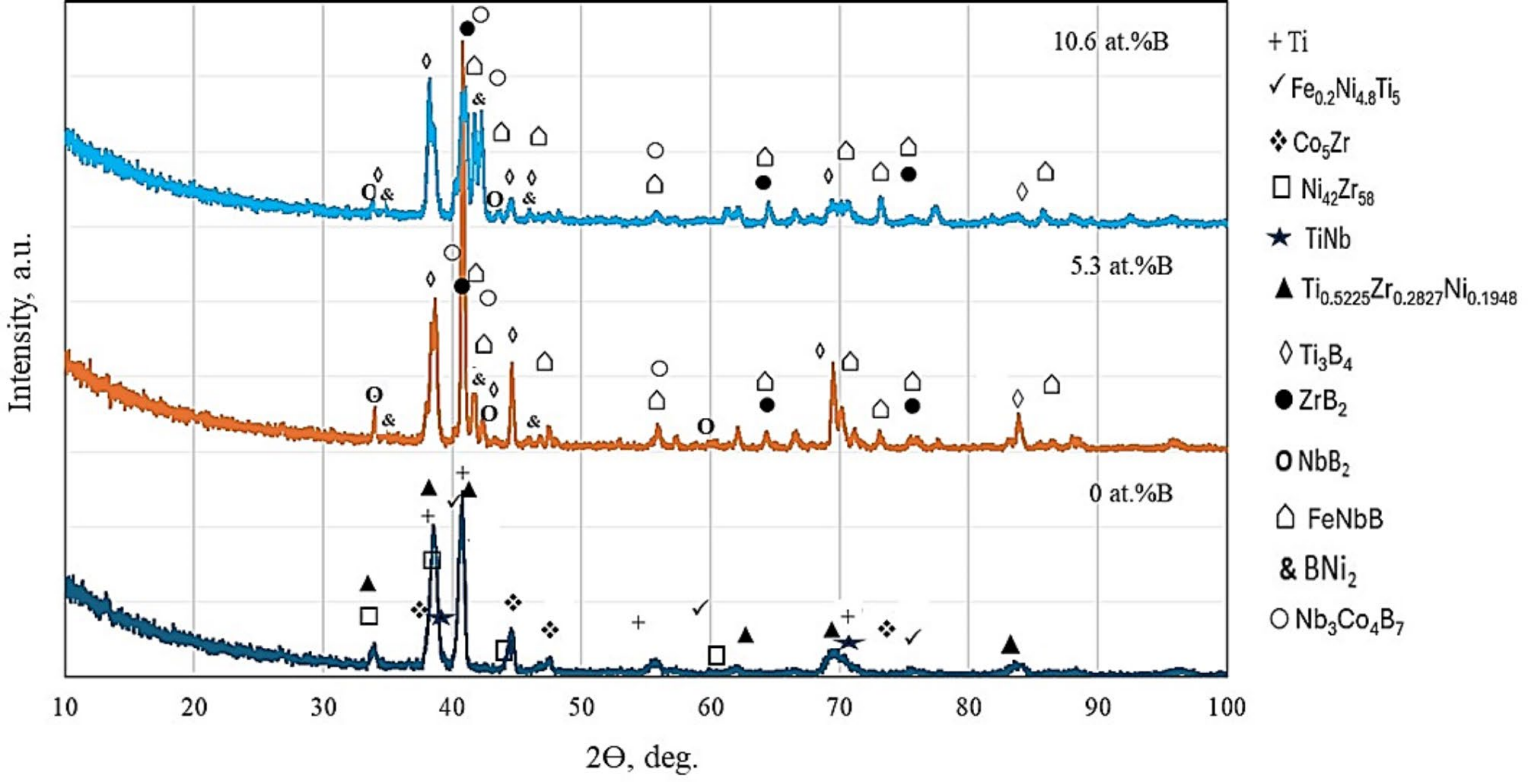
The XRD patterns of cast Ti_53.3-x_Nb_10_Zr_10_Ni_10_Co_10_Fe_6.7_B_x_ CCAs.

**Fig. 4:**
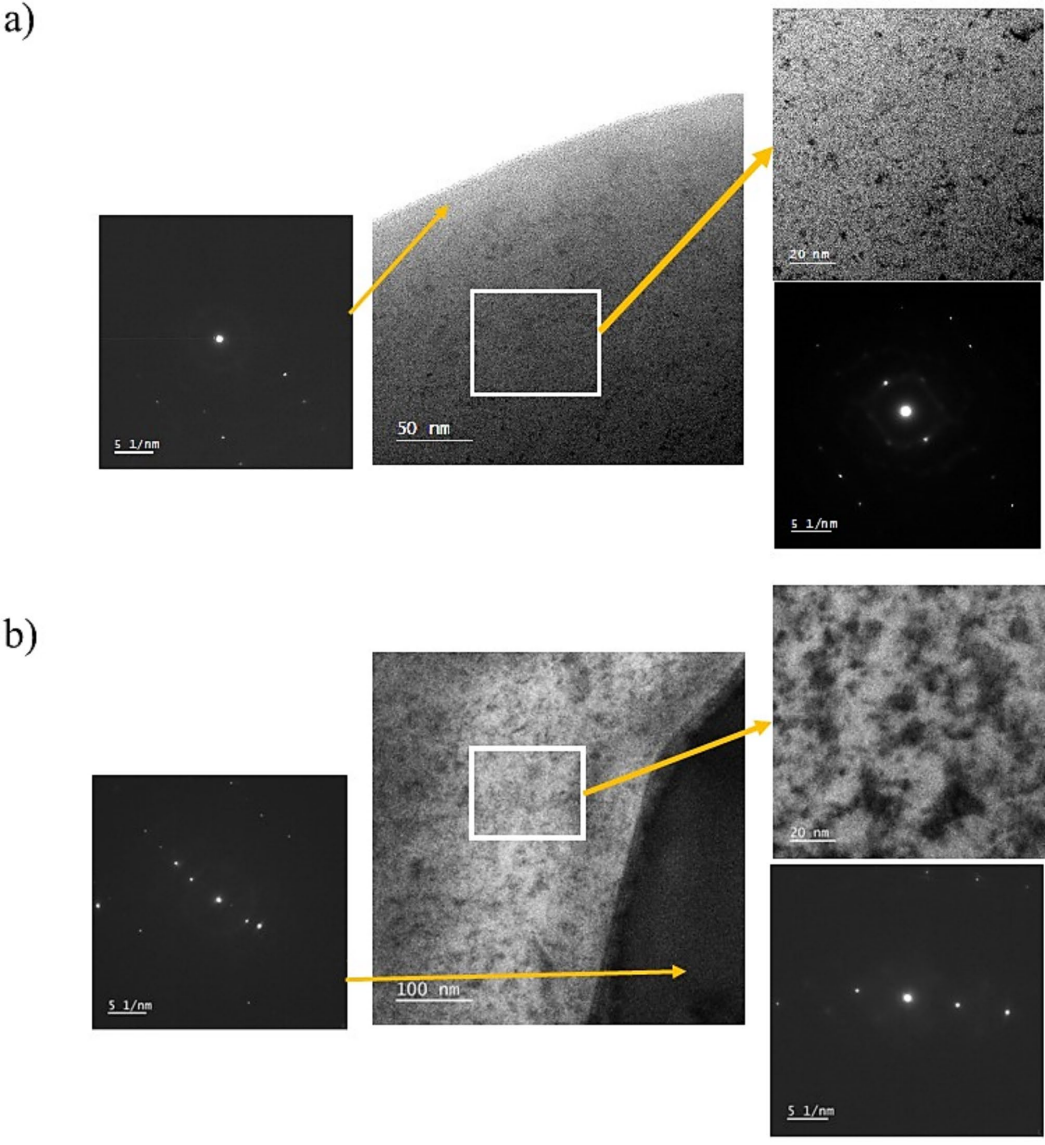
TEM images of (**a**) Ti_48_Nb_10_Zr_10_Ni_10_Co_10_Fe_6.7_B_5.3_ and (**b**) Ti_42.7_Nb_10_Zr_10_Ni_10_Co_10_Fe_6.7_B_10.6_ CCAs.

The XRD patterns of the investigated CCAs are provided in Fig. 3 and Table 2. The base alloy is composed of a cubic structure (Ni_42_Zr_58_) reported in^50^ as the main phase with some other phases that are composed of (Ti_0.5225_Zr_0.2827_Ni_0.1948_)^52^, Co_5_Zr, and others. Adding B decreased the XRD-qualitative percentage of Ni_42_Zr_58_ (from 64 to 41 at 5% B and down to 30 at 10.6 at.%), and encouraged the formation of borides. It has been reported that both Nb and Zr interact with B via the eutectic reaction thus producing ZrB_2_ and NbB_2_ in binary reactions^53,54^. Similarly, Ni and B have been reported to form borides^55^. Complex borides such as FeNbB and Nb_3_Co_4_B_7_ were also detected. According to^54,55^, these borides are expected to contribute to alloy strength. Transition metal diborides such as TiB_2_ and ZrB_2_ showed high hardness and elastic modulus. Figure 4a provides the TEM image of the cast Ti_48_Nb_10_Zr_10_Ni_10_Co_10_Fe_6.7_B_5.3_ CCAs. This TEM micrograph represents complete crystallinity. It is also clear from TEM images that microstructure coarsening occurred, and the samples became less homogeneous, with increasing the B to 10.6 at.%, Fig. 4b.

**Table 2:** Summary of XRD main data shown in Fig. 3.

Phases	Reference code	Angles	Miller indices
Ti	00-044-1294	38.4	(0 0 2)
		40.1	(1 0 1)
		53	(1 0 2)
Ni_42_Zr_58_	00-040-1035	34	(4 2 1)
		38.6	(5 1 1)
		44.5	(5 3 1)
Ti_0.5225_Zr_0.2827_Ni_0.1948_	01-087-5951	40.5	(1 1 2)
		41	(2 0 1)
		65.3	(3 0 2)
TiNb	03-065-9438	38.7	-110
		70.09	-211
Fe0.2Ni4.8Ti5	00-048-1832	42.4	(1 1 0)
		61.5	(2 0 0)
		77.5	(2 1 1)
Co_5_Zr	00-040-0863	38	(2 2 0)
		40.8	(5 1 1)
		44.8	(3 1 1)
Ti_3_B_4_	00-019-1368	35.4	(0 3 1)
		42.7	(1 2 1)
		43	(1 5 0)
ZrB_2_	34-04-0742	41	(1 0 1)
		64	(1 1 1)
		74	(2 0 1)
FeNbB	01-079-2869	41	(1 1 1)
		44.7	(2 0 1)
		46.1	(2 1 0)
Nb_3_Co_4_B_7_	00-039-0483	41.7	− 113
		42.5	− 152
		57.5	− 200
NbB_2_	01-075-0965	43.5	− 101
		33.5	− 100
		26.9	− 1
BNi_2_	00–048-1222	45.89	− 211
		35.9	− 200
		42.5	− 2

### Microstructure investigation

**Fig. 5:**
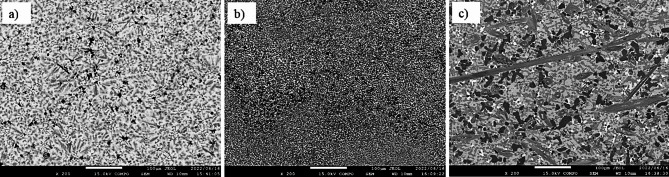
SEM micrographs of Ti_53.3-x_Nb_10_Zr_10_Ni_10_Co_10_Fe_6.7_B_x_ CCAs with B additions in at.% (**a**) 0.0, (**b**) 5.3, and (**c**) 10.6.

**Fig. 6:**
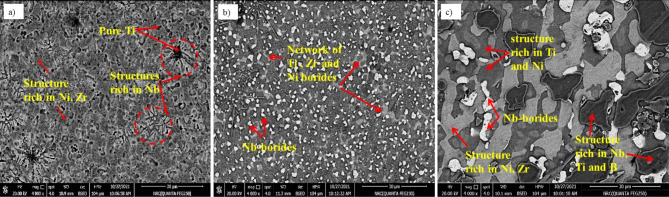
Magnified SEM micrographs of cast Ti_53.3-x_Nb_10_Zr_10_Ni_10_Co_10_Fe_6.7_B_x_ CCAs with B additions in at.% (**a**) 0.0, (**b**) 5.3, and (**c**) 10.6.

Additions of B to Ti- alloys with certain limits have been attempted with beneficial effects such as microstructure refinement in pure Ti^56^, near alpha alloys such as Ti–6Al–2Sn–4Zr–2Mo^57^, beta Ti- alloys^58^, and Ti-6Al-4V alloy^59^. Figure 5 shows the SEM micrographs of the cast Ti_53.3-x_Nb_10_Zr_10_Ni_10_Co_10_Fe_6.7_B_x_ CCAs, where their magnified images were provided in Fig. 6. It can be observed here that CCA alloy containing 5.3 at.%B has a more homogeneous microstructure than that with 10.6 at. It is also notable that the presence of B in Ti_53.3-x_Nb_10_Zr_10_Ni_10_Co_10_Fe_6.7_B_x_ CCAs led to great changes in morphology and grain size as mentioned in the literature before ^59^. The grains became equiaxed to columnar with a decrease in size by 5.3 at.% additions. However, the increase to 10.6 at.% B led to grains coarsening.

**Fig. 7:**
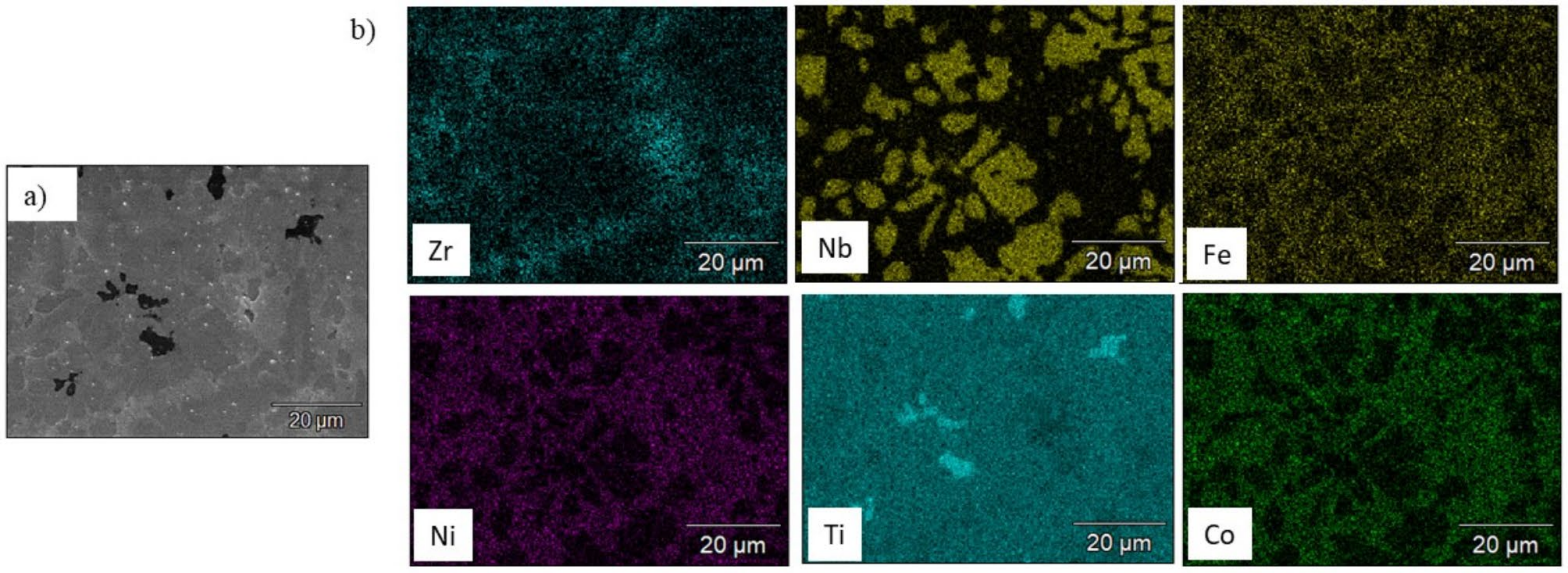
SEM micrograph (**a**) and elemental mapping (**b**) of Ti_53.3_Nb_10_Zr_10_Ni_10_Co_10_Fe_6.7_ CCA.

**Fig. 8:**
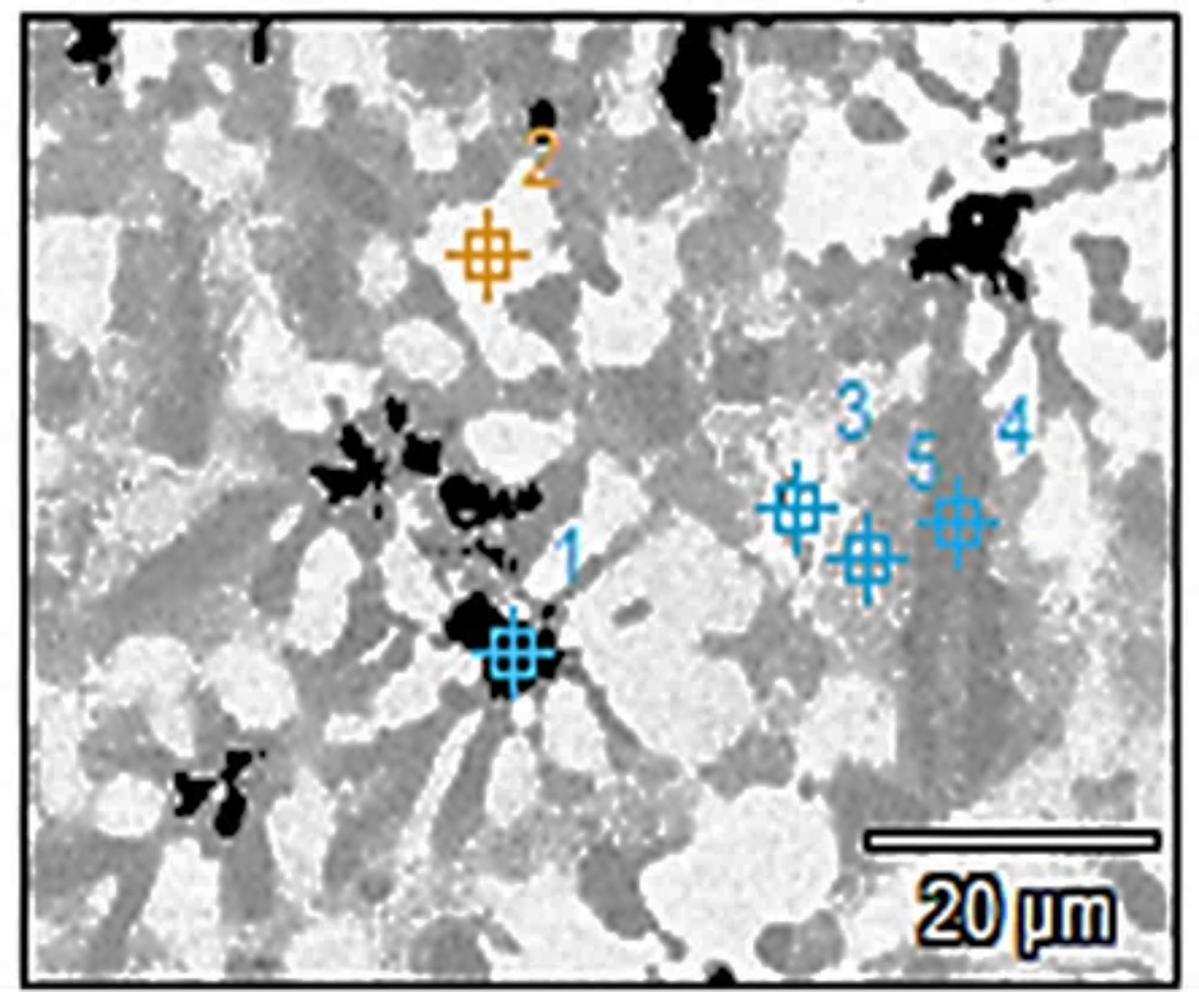
The EDX Spots locations on the Ti_53.3_Nb_10_Zr_10_Ni_10_Co_10_Fe_6.7_ CCA.

The microstructure of the obtained CCAs shown in Fig. 6 was too complex to identify the phases separately. The following EDX analysis of the prepared CCAs shows that some phases overlap thus, they cannot be distinguished using color contrast. Figure 7 shows the EDX elemental mapping of Ti_53.3_Nb_10_Zr_10_Ni_10_Co_10_Fe_6.7_ CCA. It is observed that the microstructure of this alloy contains some segregation of Ti besides the main Ni_42_Zr_58_, Fe_0.2_Ni_4.8_Ti_5,_ and Co_5_Zr phases. However, this segregation is very common in the vacuum arc melting process of HEAs and CCAs^4,5^. The corresponding EDX micrograph that locates some spots, and their analysis are shown in Fig. 8 and Table 3 respectively.

**Table 3:** EDX results of Ti_ 53.3_Nb_10_Zr_10_Ni_10_Co_10_Fe_6.7_ CCA, Fig. 8.

Spot No	Elements, at.%					
	Fe	Ni	Zr	Nb	Co	Ti
1	0.27	0.38	6.59	2.62	0.23	Bal
2	2.94	1.82	7.69	30.88	2.81	Bal
3	4.51	15.98	29.34	1.01	8.72	Bal
4	8.39	12.34	10.76	2.92	12.40	Bal
5	7.44	13.27	17.74	1.87	11.80	Bal

The dark phase in Spot (1) has high Ti, while the white phase in Spot (2) has a high Nb content. The white–grey phase in Spot (3) has high Ni and Zr (possibly Ni_42_Zr_58_), while the dark gray phase in Spot (4) and the lighter gray phase in Spot (5) have high Zr, Ni, Co, and Fe. Spots 4 and 5 may contain (Co_5_Zr and FeNiTi phases overlapping each other).

**Fig. 9:**
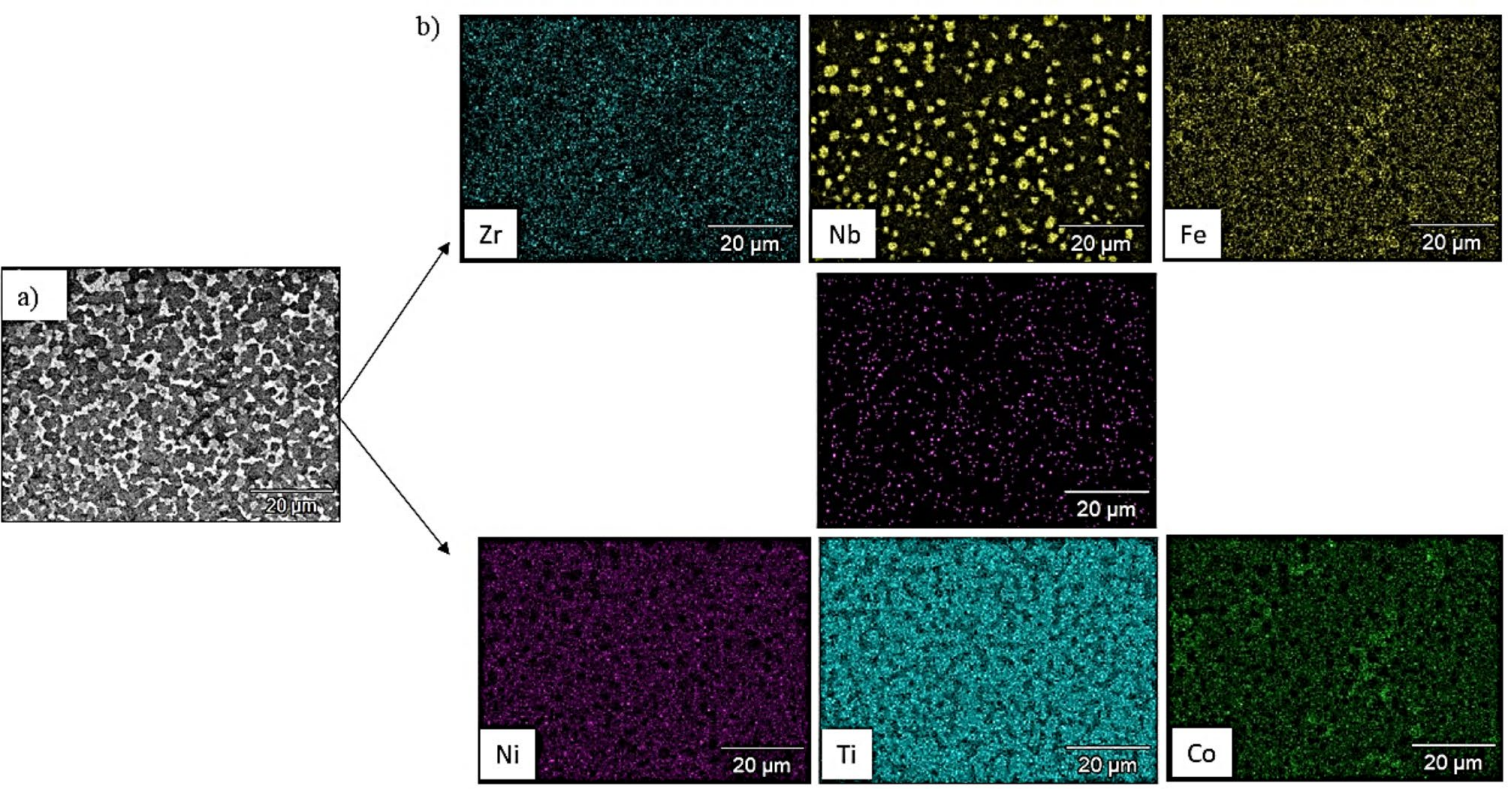
(**a**) FEM micrograph and (**b**) elemental mapping of Ti_48_Nb_10_Zr_10_Ni_10_Co_10_Fe_6.7_B_5.3_ CCA.

**Fig. 10:**
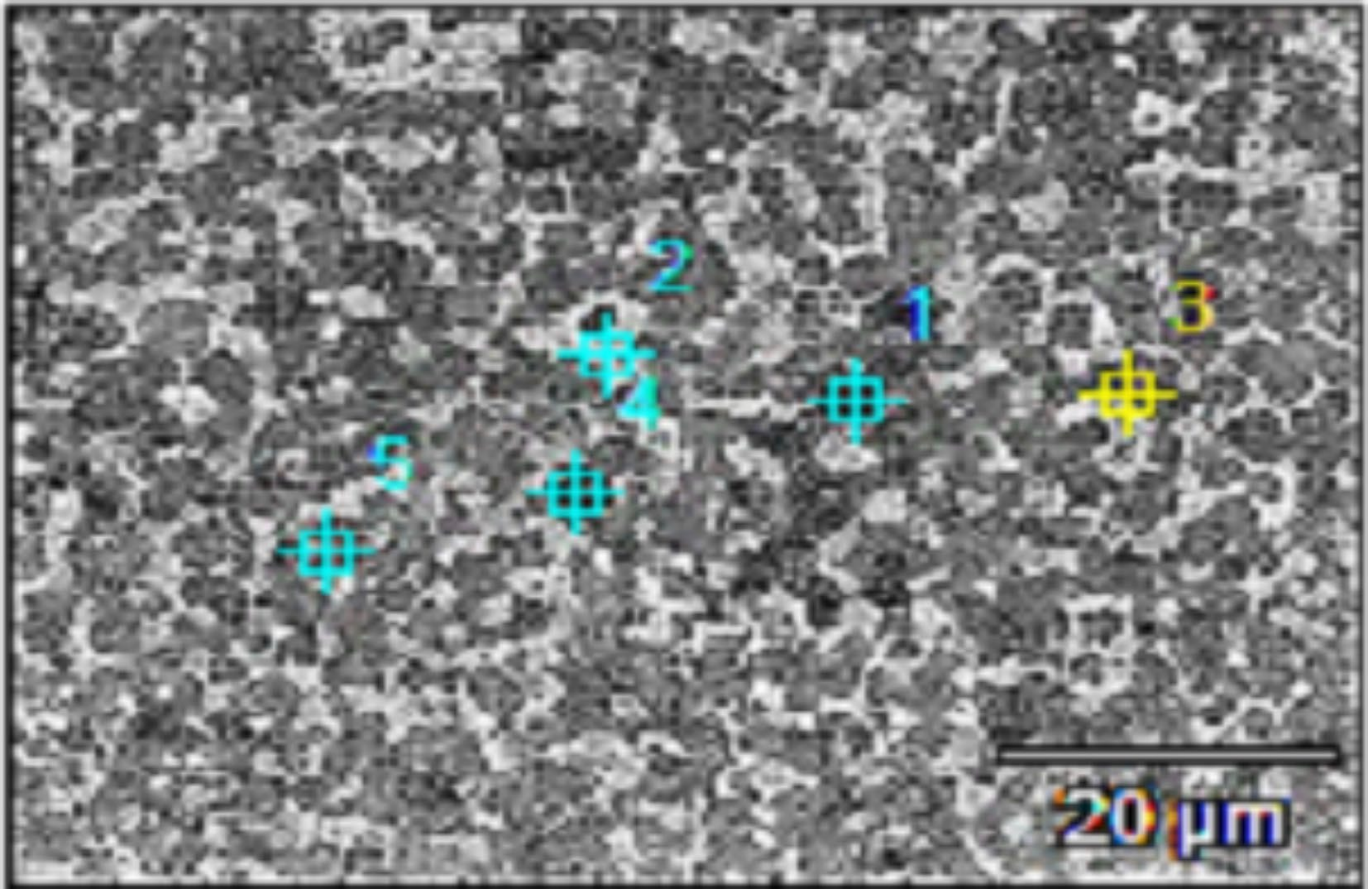
The EDX Spots locations on the Ti_48_Nb_10_Zr_10_Ni_10_Co_10_Fe_6.7_B_5.3_ CCA.

The elemental mapping of Ti_48_Nb_10_Zr_10_Ni_10_Co_10_Fe_6.7_B_5.3_ CCA, is presented in Fig. 9. This map emphasizes the good distribution of B. Figure 10 and Table 4 present the EDX spots location and their analysis respectively. The dark phase in Spot (1) has high B, Ni, Co, and Fe, while the white–gray phase in Spot (2) has high Zr and Ni. The white phase in Spot (3) has high B and Nb (might be phase NbB_2_ as discussed in the XRD section) along with some pure Nb. The grey phase in Spots (4) and (5) contains high B, Zr, and Ni (might be ZrB_2_ with Ni_42_Zr_58_). Generally, a good distribution of the phases could be obtained in 5.3 at.% B CCA, based on Fig. 9, except for some segregation of Nb as observed in spot 2. In this map, a network of borides (Ti, Ni, Nb, Zr, and Fe) can be observed which is good in terms of the expected positive effects of these intermetallic phases on the mechanical properties.

**Table 4:** EDX results of Ti_48_Nb_10_Zr_10_Ni_10_Co_10_Fe_6.7_B_5.3_ CCA, Fig. 10.

Spot No	Elements, at.%						
	B	Fe	Ni	Zr	Nb	Co	Ti
1	7.88	13.4	14.44	3.10	1.52	21.29	Bal
2	5.33	7.12	13.68	26.49	8.62	9.09	Bal
3	3.10	1.19	1.16	6.33	55.22	1.32	Bal
4	6.55	7.58	12.32	14.14	5.94	11.93	Bal
5	6.74	8.23	12.31	12.95	5.68	11.97	Bal

**Fig. 11:**
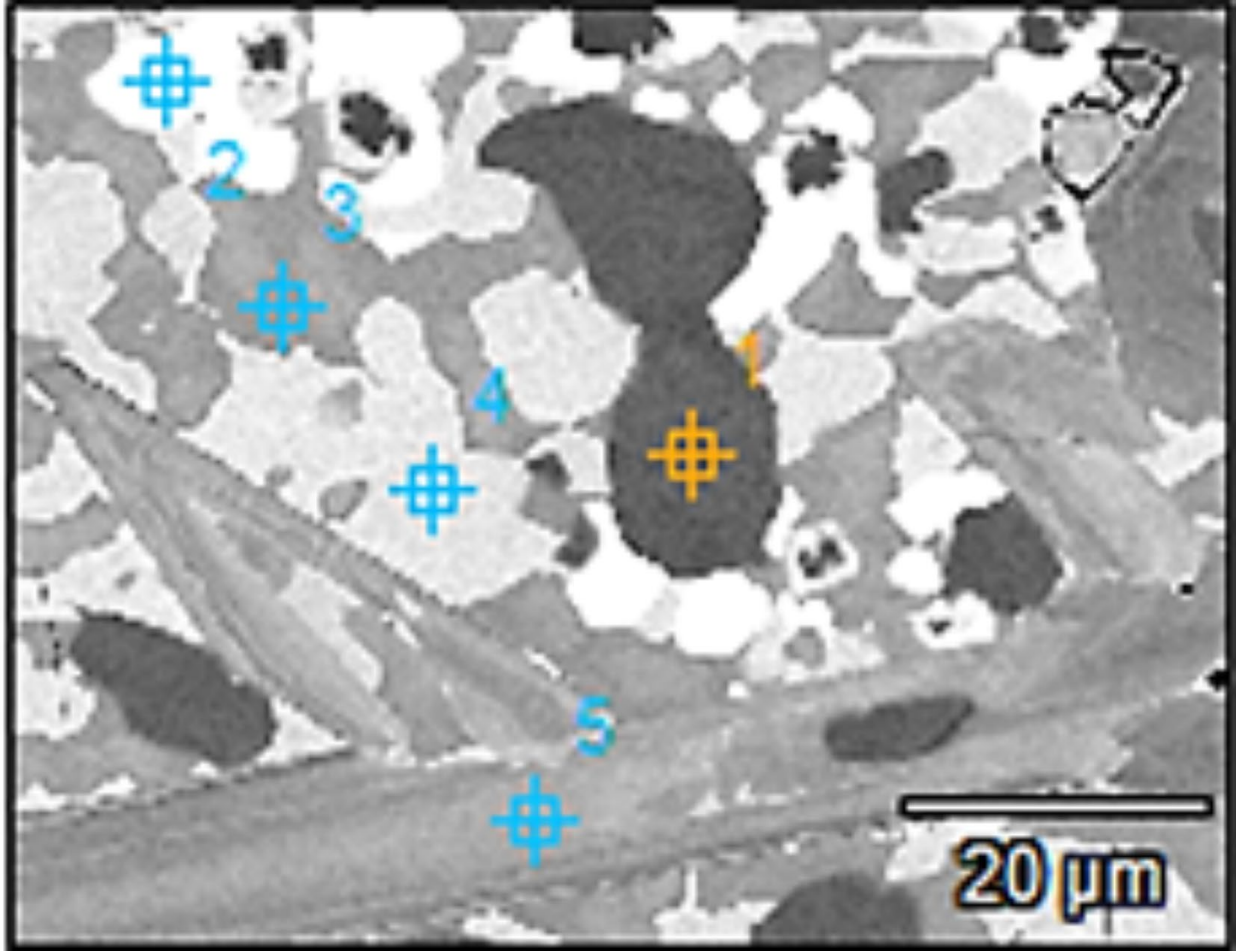
EDX spots taken in Ti_42.7_Nb_10_Zr_10_Ni_10_Co_10_Fe_6.7_B_10.6_ CCA.

Figure 11 and Table 5 represent an example of the EDX analysis of Ti_42.7_Nb_10_Zr_10_Ni_10_Co_10_Fe_6.7_B_10.6_ CCA. From this example, the black phase (Spot #1) has Co, Ni, Fe, and B content. The white phase (Spot #2) is mainly Nb with some B. The dark grey phase (Spot # 3) has high Co, Zr, Ni, and Fe. The light grey phase of Spots #4 and #5, has Nb and B. Compared to Ti_48_Nb_10_Zr_10_Ni_10_Co_10_Fe_6.7_B_5.3_ CCA, inadequate distribution of the phases along with coarser grains can be observed in Ti_42.7_Nb_10_Zr_10_Ni_10_Co_10_Fe_6.7_B_10.6_ CCA.

**Table 5:** EDX results of Ti_42.7_Nb_10_Zr_10_Ni_10_Co_10_Fe_6. 7_B_10.6_ CCAs, Fig. 11.

Spot No	Elements, at.%						
	B	Fe	Ni	Zr	Nb	Co	Ti
1	7.39	13.1	14.5	3.96	2.21	22.03	Bal
2	2.45	1.37	0.78	5.1	62.17	1.07	Bal
3	6.38	7.49	12.87	13.46	4.75	12.35	Bal
4	5.49	7.76	13.74	25.15	9.39	9.41	Bal
5	4.53	0.15	0.19	3.16	57.98	0.38	Bal

### Mechanical properties of Ti_53.3-x_Nb_10_Zr_10_Ni_10_Co_10_Fe_6.7_B_x_

As a consequence of the observed influence of B on the microstructure of the CCAs, the mechanical properties were considered.

#### Hardness

Most research works focused on improving the hardness of beta-Ti alloys through thermomechanical effects. Using more advanced digital control systems to precisely select processing parameters, such as heat treatment and complex microstructure building, could be a future development path^55,60^. In the current experiments, significant improvement was noticed in the hardness of CCAs by adding 5.3 at.% B and 10.6 at% in 20% and 30%, respectively. Hardness increased from 502 to 606 HV with 5.3 at.% B and to 648 HV with 10.6 at.%B. This improvement in the hardness with B addition can be principally owed to the formation of new B-based intermetallic phases, shown in XRD results. These boride phases such as BNi_2_, NbB_2_, Ti_3_B_4_, TiB_12,_ and ZrB have been reported to be stable strengthening phases^54,55^. Consequently, increasing the B content supports formation of these phases and hence increases the alloys’ hardness.

#### Wear resistance

The wear mechanisms generally occurring in Ti-alloys include oxidation, adhesive, abrasive, and layered wear^61,62^. On the other hand, different loading sliding velocities, matrix materials, and ambient temperature conditions alter the wear mechanisms^63^. The sliding wear resistance is proportional to the alloy’s hardness, according to Archard’s laws. Furthermore, the COF can influence the material’s wear resistance. Generally, a low friction coefficient benefits the material’s wear resistance. Some researchers modified beta-Ti to reduce the COF on the material surface to 0.42 in Ti-13Zr-13Nb-0.5B alloy^26^.

**Fig. 12:**
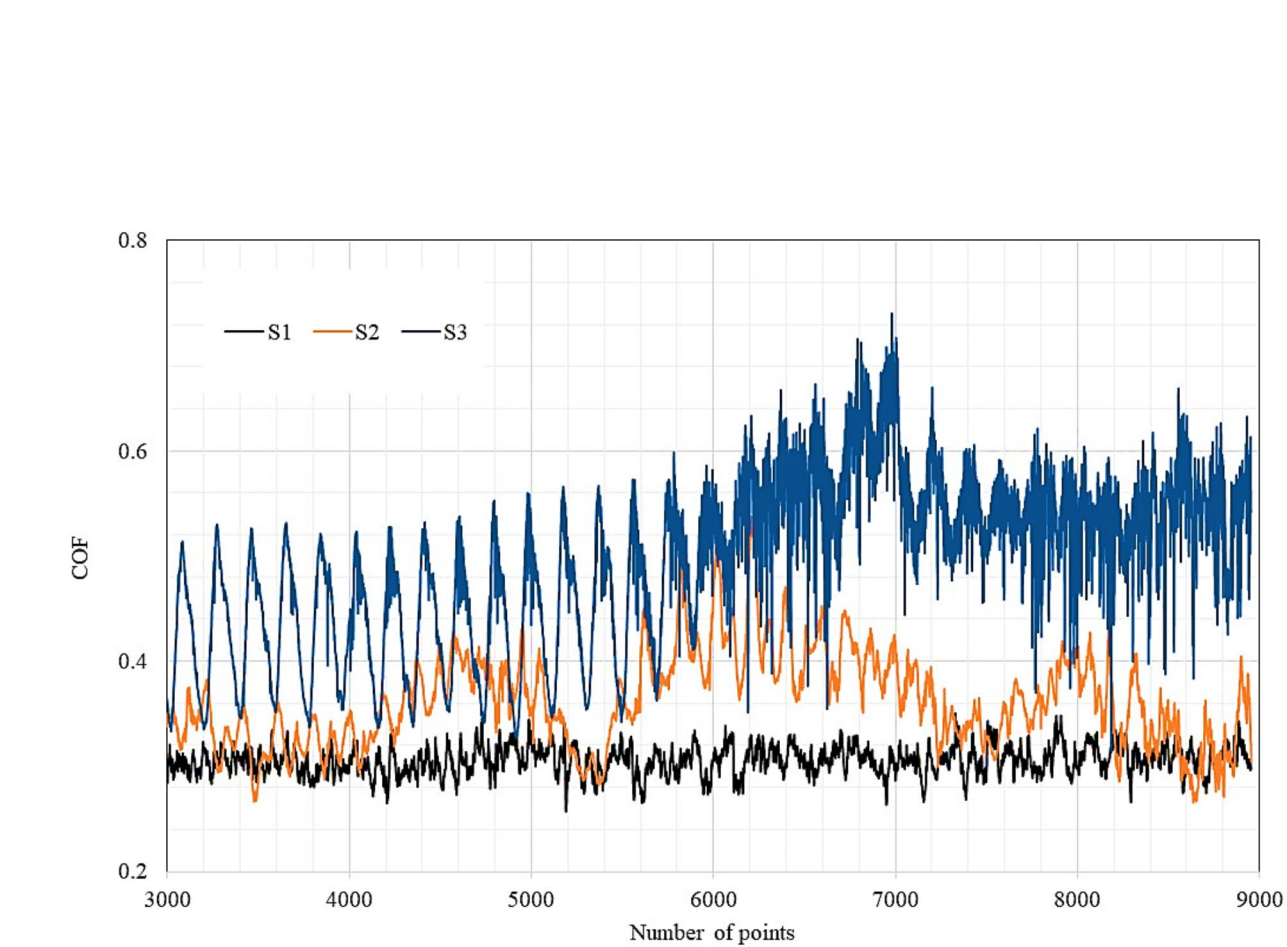
COF of Ti_53.3-x_Nb_10_Zr_10_Ni_10_Co_10_Fe_6.7_B_x_ CCAs.

The coefficients of friction (COF) values were recorded for the current tests, as shown in Fig. 12. The CCAs without B (S1) recorded a COF of about ~ 0.3. Alloying with 5.3 at.% B showed almost no change in the value of COF (~ 0.33) while increasing the B to 10.6 at.% led to a significant increase in the COF to (~ 0.5). Regarding the wear rate (WR) of the tests performed at 0.9 km sliding distance, speed of 1 m/s, and a load of 10 kg, increasing the B content decreased the WR from ~ 0.13 to ~ 0.03 with 5.3 at.% B addition and then to ~ 0.004 at 10.6 at.% B. This results in line with the hardness values, which also increased with B addition, due to the formation of boride phases.

Here, it is worth noting that B addition decreased the WR while increasing the friction coefficient. This seems contradicting to the previous literature^53,55^, where it was mentioned that improving wear resistance relies on decreasing COF. This can be explained through the recent Kato et al. work^61^, where it was stated that multiphase alloys such as Fe, Cu, and Al systems have phases that differ in hardness and oxidation behavior. When these multiphase surfaces are rubbed in the air by another solid surface, the generated oxides differ in their types and influence the friction coefficient value. However, the general behavior of the alloy during friction in the classical alloying systems is related to the principal element (Fe, Al, etc.). In the current investigation, the process is more complex since we have HEAs with multi-principal constituents (Ti, Nb, Zr, Ni, Co, and Fe) where the value of COF cannot be related to a single phase. Some of these oxides can decrease COF, while others may exhibit negative effects. This is evident following the behavior of COF curves in Fig. 12, in which an almost stable trend is observed in S1 while adding B resulted in the formation of other compounds and phases, affecting COF stability during the wear test. According to Luo et al.^64^, the tribological performance of CCAs in different environments still needs more research to widen the practical application of CCAs as a coating material.

#### Young’s modulus

It has been reported that the dispersion of hard particles, such as carbides or borides can significantly raise Young’s modulus of Ti alloys^35^. It is worth noting that in CCAs, mixing the main elements in equimolar proportion, results in lattice distortion, leading to an increase in yield strength due to the lattice’s higher resistance to dislocation movement^65^. Due to strong solid solution strengthening, equiatomic TiNbZr, for example, combines good strength and excellent ductility^35,65^. TiNbZr alloys have Young’s modulus of about 48–80 GPa, which is significantly lower than that of Ti-6Al-4V (110 GPa), 316L stainless steel (200 GPa), and Co-Cr alloys (210–232 GPa)^65^. The Young’s modulus in the current work was measured by physical methods (ultrasonic) due to the small size of the prepared samples. The measured values of Young’s modulus were in good agreement with the hardness values obtained for the different alloys. Adding B increased Young’s modulus from 141 GPa (without B) to 195 GPa and 260 GPa with 5.3 and 10.6 at.%B, respectively. These results were expected due to the strengthening boride phases observed in the XRD patterns of the different alloys. These five phases: BNi_2_, NbB_2_, Ti_3_B_4_, TiB_12_, and ZrB contributed to strengthening the prepared CCAs.

### Assessment of impact toughness

To assess the impact toughness of the three CCAs, microhardness was measured for the different samples. According to the early investigation by Nihara et al.^66^, the impact toughness can be estimated using Eq. (14) as long as the cracks are observed clearly during the microhardness test. Although this equation is more applicable to hard composites and intermetallic alloys, it has been used to estimate the fracture toughness of brittle samples^67^. The crack geometry, for which the relation between fracture toughness, crack size, and other relevant parameters given by Eq. (14) is known as “Palmqvist crack”.

14 $${K}_{IC}=0.035* {\Phi }^{-\frac{3}{5}}*H*\left(\frac{d/2}{\sqrt{l}}\right)*{\left(\frac{H}{E}\right)}^{-\frac{2}{5}}$$

where K_IC_ is the fracture toughness, Φ is a constraint factor taken from^68^, H is the average microhardness, E is Young’s modulus, d is the average diagonal length of indentation, and L is equal to the total length of the Palmqvist crack. Table 6 lists the average microhardness values, indentations, and the corresponding K_IC_ wherever a crack could be detected. It is notable that sample “S3” which has 10.6 at.% B, and exhibited the highest hardness and Young’s modulus showed brittle behavior, indicated by the indentation-induced cracking. On the other hand, sample “S2” with a moderate amount of B (5.3 at.%), where a more homogeneous microstructure was observed, showed no cracks.

**Table 6:** Calculated fracture toughness of the prepared CCAs.

At.% B	Hv, 9.8 N	Indentation	K_IC_, MPa.(m^1/2^)
0.0	538.2 ± 14		No cracks
5.3	612.4 ± 13.6		No cracks
10.6	660 ± 20		~ 3.3

## Conclusions

The current work investigated the effect of B additions (5.3 and 10.6 at.%) on the metallurgical characteristics of a novel cast Ti_53.3-x_Nb_10_Zr_10_Ni_1__0_Co_10_Fe_6.7_B_x_ CCAs. It was concluded that:

1. Adding B to Ti_53.3_Nb_10_Zr_10_Ni_10_Co_10_Fe_6.7_ CCAs decreased the total VEC thus enhancing the stability of the BCC structure, which is the main phase.
2. Formation of BNi_2_, NbB_2_, Ti_3_B_4_, TiB_12,_ and ZrB intermetallic phases caused second phase strengthening.
3. The hardness increased from 502 to 606 HV with 5.3 at.%B and to 648 HV with 10.6 at.%B. Consequently, enhanced wear properties were obtained.
4. Young’s modulus increased from 141 to 195 GPa and 260 GPa with 5.3 and 10.6 at.% B, respectively.
5. Increasing the amount of B to 10.6 at.% led to brittleness that was indicated by cracking during the microhardness test. Therefore adding 5.3 at.% B is recommended to obtain homogeneous CCAS with enhanced strength.

## Data Availability

The datasets used and/or analyzed during the current study are available from the corresponding author upon reasonable request.
